# Supplementary material for: The genome sequence of a black lacewing, Nothochrysa capitata (Fabricius, 1793) (Neuroptera: Chrysopidae)
Source: Wellcome Open Res. 2025 Nov 24;10:657. [Version 1] doi: 10.12688/wellcomeopenres.25199.1 (PMC12805232; doi:10.12688/wellcomeopenres.25199.1)
Supplement: Peer review history

# Reviewer report: Reviewer response for version 1

**DOI:** 10.21956/wellcomeopenres.27775.r143569

**Published:** 2026-01-14

**Authors:** Jan Veenstra

**Affiliations:** 1 University of Bordeaux, Pessac, France

This is yet another high quality genome assembly from Welcome Open Research. It is sometimes annoying that you have to write something, as in general the comments could be as simple as: "Great work, thanks a lot for doing this". This time I actually have a, really minor, comment to make. So here it comes:

 The "but" in the sentence: "No Chrysopidae from Europe have at this moment been assessed for risk of extinction by the IUCN, but this common, well researched species with a wide distribution has not been flagged as a conservation concern in the literature." seems weird to me, as I do not see a contrast between the first and second part of the sentence.

Are sufficient details of methods and materials provided to allow replication by others?

Yes

Is the rationale for creating the dataset(s) clearly described?

Yes

Are the datasets clearly presented in a useable and accessible format?

Yes

Are the protocols appropriate and is the work technically sound?

Yes

Reviewer Expertise:

Insect neuropeptides

I confirm that I have read this submission and believe that I have an appropriate level of expertise to confirm that it is of an acceptable scientific standard.

# Reviewer report: Reviewer response for version 1

**DOI:** 10.21956/wellcomeopenres.27775.r143567

**Published:** 2026-01-13

**Authors:** Matsapume Detcharoen

**Affiliations:** 1 Division of Biological Science, Faculty of Science, Prince of Songkla University, Hat Yai, Songkhla, Thailand, Hat Yai, Thailand

This note reports a high-quality genome assembly of Nothochrysa capitata, generated using PacBio HiFi and Hi-C data as part of the Darwin Tree of Life project. The authors produce a chromosome-level assembly for one haplotype, including identification of the W and Z sex chromosomes, and provide extensive quality metrics demonstrating high completeness and consensus accuracy.

 The manuscript is clearly written. Assembly quality is high, with BUSCO completeness >97% and transparent reporting of curation steps.

 A brief explanatory sentence on the chromosome assignment benchmark in Table 4 between 78.72% vs 90% target would improve clarity for readers.

Are sufficient details of methods and materials provided to allow replication by others?

Yes

Is the rationale for creating the dataset(s) clearly described?

Yes

Are the datasets clearly presented in a useable and accessible format?

Yes

Are the protocols appropriate and is the work technically sound?

Yes

Reviewer Expertise:

genomics, applied biology

I confirm that I have read this submission and believe that I have an appropriate level of expertise to confirm that it is of an acceptable scientific standard.
